# Study on the Zinc Nutritional Status and Risk Factors of Chinese 6–18-Year-Old Children

**DOI:** 10.3390/nu15071685

**Published:** 2023-03-30

**Authors:** Jiaxi Lu, Huidi Zhang, Wei Cao, Shan Jiang, Hongyun Fang, Dongmei Yu, Lichen Yang

**Affiliations:** Key Laboratory of Trace Element Nutrition, National Health Commission of the People’s Republic of China, National Institute for Nutrition and Health, Chinese Center for Disease Control and Prevention, Beijing 100050, China

**Keywords:** zinc, children, Zn deficiency, anemia

## Abstract

Zinc is an essential micronutrient that is involved in several metabolic processes, especially children’s growth and development. Although many previous studies have evaluated the zinc nutritional status of children, there are very few reports on children aged 6–18 years old. Furthermore, there are few reports on children’s zinc nutrition status based on the Chinese population. According to WHO data, the prevalence of zinc deficiency in Asian countries is rather high and has resulted in high child mortality. In this study, we aimed to comprehensively assess zinc nutritional status and the prevalence of zinc deficiency among children aged 6–18 years in China based on nationally representative cross-sectional data. Subgroup comparisons were made under possible influencing factors. The potential risk factors of zinc deficiency were also discussed. A total of 64,850 children, equally male and female, were recruited from 150 monitoring sites in 31 provinces through stratified random sampling from China National Nutrition and Health Survey of Children and Lactating Mothers (CNNHS 2016–2017). Median and interquartile intervals were used to represent the overall zinc concentration levels and different subgroups. A Chi-square test was used to compare serum zinc levels and the prevalence of zinc deficiency in children under different group variables. In order to study the influencing factors of zinc deficiency, multiple logistic regression was utilized. It was found that the median concentration of serum Zn was 88.39 μg/dL and the prevalence of Zn deficiency was 9.62%. The possible influence factors for Zn deficiency were sex, anemia, nutritional status, city type and income. By conducting a subgroup analysis of the factors, it was found that males; those with anemia, stunting and low income; and children living in rural areas have a higher risk of Zn deficiency. This study offers a comprehensive analysis of Zn nutritional status among Chinese children, which provides reliable data for policy formulation to improve the zinc nutrition status of children.

## 1. Introduction

Zinc (Zn) is the second most abundantly traced element in the body [1]. It is an essential cofactor required for a number of functions, for example, it catalyzes more than 100 enzymes’ activity, facilitates protein folding, and regulates gene expression [2]. According to a report by the World Health Organization (WHO) [3], Zn deficiency due to malnutrition ranks 11th among the major risk factors contributing to the global burden of disease. Early in the 20th century, severe zinc deficiency was identified as a syndrome marked by anorexia, hypogonadism, immune system impairment, skin abnormalities, and cognitive dysfunction [4]. In 1926, Lutz et al. used the dithizone technique for the first time to estimate the total zinc content of a 70 kg man to be 2.2 g. By 2004, the International Zinc Nutrition Consultative Group (IZiNCG) proposed a method for the comprehensive assessment of human zinc nutritional status [5]. More and more evidence shows that zinc may play an important role in a series of metabolic and chronic diseases in the body. Despite the rarity of severe zinc deficiency, mild-to-moderate zinc deficiencies are relatively widespread worldwide. Zn deficiency affects around one-third of the world’s population, with estimates varying from 4% to 73% depending on the subregion, with the vast majority occurring in developing countries in Africa and Asia [6,7]. In 2002, 1.4% (0.8 million) of fatalities were linked to zinc deficiency [3]. It has been reported that zinc deficiency has a greater impact on children and adolescents, which can cause growth retardation and even the death of children [8,9,10]. In 2011, 116 000 child fatalities were attributable to zinc deficiency, which affects an estimated 17% of the world’s population [11,12]. The main indicator for determining zinc insufficiency in people is serum zinc.

The lack of concern about zinc deficiency in children has been a widespread obstacle. Understanding children’s zinc nutritional status and zinc deficiency under different nutritional conditions is crucial for the future of global health policy. In this study, we aimed to conduct a comprehensive assessment of zinc nutrition in Chinese children aged 6–18 years based on a nationally representative cross-sectional study. The deficiency of zinc in whole and different subgroups was also explored. At the same time, we also discuss the possible risk factors of zinc deficiency.

## 2. Materials and Methods

### 2.1. Subjects

All the subjects were enrolled from the China National Nutrition and Health Survey of Children and Lactating Mothers (CNNHS 2016–2017), which is a national representative cross-sectional survey [13]. All the participants were selected from a total of 150 monitoring sites in 31 provinces across the country by stratified, multistage and random selection methods. Children aged 6–17 y were selected from schools in each monitoring site, half boys and half girls. Thus, the results are nationally, city type and regionally representative. 

Biological index values below the detection limit, samples with incomplete questionnaire responses, and poor blood quality (such as hemolysis) were also excluded. In this study, 64,850 samples were finally included for analysis. All participants and their guardian were given their written consent after being fully informed. The Ethics Review Board of NINH, China CDC, approved the protocol (No. 201614).

### 2.2. Basic Information and Sample Collection

Basic demographic data (including age, sex, address, etc.) were gathered by consistently trained investigators using a standardized questionnaire. Additionally, the regional types of the subjects were classified accordingly [14]. Anthropometric measures were also taken by skilled medical personnel using defined protocols. Measurements of height and weight are part of the physical examination [15]. Weight and height were measured to the nearest 0.1 kg and centimeter using the Seca 877 electronic flat scale and the Seca 213 portable height bar gauge, respectively. During the measurements, the subject was required to remove their shoes, hats, and coats, and females untied their braids to ensure the accuracy of measurements. Body mass index (BMI) is calculated as weight (kg)/height (m^2^) squared. The nutritional status of children can be divided into stunting, wasting, normal, overweight and obesity. Stunting and wasting were defined according to *the Chinese screening standards for malnutrition of school-age children and adolescents (WS/T 456—2014)* ^,c^ [16]. Additionally, the overweight and obesity of children were defined according to *the Chinese screening standards for malnutrition of school-age children and adolescents (WS/T 586—2018)* [17].

After at least 10 h of fasting, venous blood was drawn in the morning. Each blood sample was separated into an anticoagulant tube and a serum separator tube. Between 20 and 30 min after the blood was drawn, serum separator tubes containing blood samples were immediately centrifuged at 1500× *g* for 15 min. Afterward, the sera were separated and stored at −70 °C for further testing. 

### 2.3. Serum Zn and Hemoglobin (Hb) Laboratory Analyses and Evaluation Standards

Inductively coupled plasma mass spectrometry (ICP-MS, PerkinElmer, NexION 350, Waltham, MA, USA) was used to detect the amounts of zinc. The precision and accuracy of the analysis were checked with the quality control samples (Seronorm, Level-2, Billingstad, Norway). Zn deficiency was defined as a serum Zn concentration lower than 70 μg/dL for girls and 74 μg/dL for boys according to the recommendation of The International Zinc Nutrition Consultative Group (IZiNCG) [18]. 

Hemoglobin was detected using a Sysmex XE-2100 hematology analyzer (Sysmex Corporation, Kobe, Japan) [19]. Additionally, anemia was defined according to the method of anemia screening (see [20]), as a Hb level lower than 115 g/L for ages 6–12 y, 120 g/L for ages 12–15 y in boys and in 15–18 y girls, 130 g/L for ages 15–18 y in boys. Because the Hb value is affected by the altitude of the long-term living area and increases with the rapid rise of altitude, we adjusted the hemoglobin value of people living at an altitude above 1000 m for more than half a year.

### 2.4. Data Analyses

For all data cleaning and analysis, SAS 9.4 (SAS Institute Inc., Cary, NC, USA) was utilized. Finally, the serum zinc content was represented as the median and interquartile range (IQR) for an abnormal distribution, and Kruskal–Wallis was used to compare the differences between the groups. The prevalence of serum zinc deficiency in children with various features was compared using the chi-square test. The multivariate logistic regression model was used to evaluate the variables affecting zinc nutrition. The value for statistical significance was set at *p* less than 0.05.

## 3. Results

### 3.1. Serum Zinc Concentration of Children 

The serum Zn concentrations of children are shown in Table 1. A total of 64,850 children had a median Zn level of 88.39 μg/dL and the interquartile interval ranged from 80.00 to 98.80 μg/dL. By comparing the serum zinc levels among different subgroups, it was found that the distribution differences of serum zinc according to sex, age group, anemia status, nutritional status, city type, district and income were statistically significant. In sex groups, females (88.00 μg/dL) had a lower Zn level than males (89.00 μg/dL). Children in the 12–18-years-old category (89.00 μg/dL) had a higher Zn concentration than 6–11-year-olds (88.00 μg/dL). There was a large difference in zinc concentration among children with anemia (86.40 μg/dL) and without anemia (88.84 μg/dL). Among the different nutritional statuses, zinc level gradually increased from stunting (86.00 μg/dL) to obesity (91.00 μg/dL). Meanwhile, there were statistical differences between the two comparisons, except that there was no difference in zinc levels between wasting and normal. In the comparation of city type, Zn level was higher in children who live in a city (89.70 μg/dL) than in children who live rurally (87.30 μg/dL). From east to west, Zn concentration gradually decreased from 90.59 μg/dL to 86.00 μg/dL. Additionally, there was a statistical difference between the comparison of both districts. Similarly, with the reduction in family income, the zinc level gradually decreased. Children who live with an income of less than CNY 7500 had the lowest Zn level, 86.00 μg/dL, while children who live with an income greater than CNY 20,000 had the highest Zn level, 89.00 μg/dL. The Zn concentration in children with a family income lower than CNY 12,500 yuan was significantly different to children with a family income of CNY 12,500~20,000 and higher than CNY 20,000.

### 3.2. The Prevalence of Zinc Deficiency in 6–18-Year-Old Chinese Children 

The prevalence of zinc deficiency analysis in Chinese children is shown in Table 2. A total of 6320 children were identified through screening as having a zinc deficiency. Additionally, the prevalence of Zn deficiency was 9.62% (95%CI 9.22–10.00%). Consistent with the distribution of zinc level, the difference in zinc deficiency among different subgroups is also statistically significant. In the comparison of sex groups, males (11.14%) had a higher prevalence of Zn deficiency than females (7.86%). The difference in Zn deficiency according to age group was consistent with that of Zn concentration. Children aged 6–11 y (10.44%) suffered with zinc deficiency more than those aged 12–18 y (8.85%). Having anemia also increased the risk of zinc deficiency; children with anemia had a higher prevalence of Zn deficiency than children without anemia (10.88% vs. 9.54%). Contrary to the trend of zinc concentration, the prevalence of zinc deficiency gradually increased with nutritional status from stunting (13.55%) to obesity (8.02%). There was no difference in zinc deficiency between the overweight and obesity groups. However, there were statistical differences in zinc deficiency rates among the other two comparisons. Children in rural areas (10.92%) had a higher prevalence of Zn deficiency compared to children living in cities (8.20%). In the comparison of districts, different from the distribution of serum zinc levels, children who live in a central area (12.36%) had the highest prevalence of zinc deficiency. Additionally, children who live in an eastern area (6.15%) had the lowest prevalence of Zn deficiency. Regional statistical differences in the prevalence of zinc deficiency in children were evident in the different groups. The zinc deficiency rate decreased significantly from 13.11% in the children with a family income lower than CNY 7500 group to 6.88% in the income greater than CNY 20,000 group. Except for the comparison of Zn deficiency between the family income less than CNY 7500 and CNY 7500 7500~<12,500 groups, there were significant differences between the other groups.

### 3.3. Multivariate Logistic Regression Model for Risk Factors Associated with Zn Deficiency in Chinese Children

An assessment of the potential risk factors associated with Zn deficiency is shown in Table 3. Among the classification variables, sex, anemia, nutritional status, city type and income were all associated with Zn deficiency. Compared with females, males had a higher risk of Zn deficiency, and the OR value for males was 1.552 (95% CI 1.410–1.709, *p* < 0.001). The OR value of Zn deficiency in people with anemia was 1.260 (95%CI 1.130–1.405, *p* < 0.001) compared with that of people without anemia. Compared with people in the city, the OR value of Zn deficiency in people who live in rural areas is 1.239 (95%CI 1.124–1.366, *p* < 0.001). In different the subgroup of nutritional status, the OR value, in comparison with normal people, was 1.443 for stunting (95%CI 1.190–1.750, *p* < 0.001), 0.881 for overweight (95%CI 0.808–0.960, *p* = 0.004), and 0.776 for obesity (95%CI 0.702–0.857, *p* < 0.001).

### 3.4. Association between Anemia and Zinc Deficiency in Different Groups

Based on the findings in Table 3, the relationship between anemia and zinc deficiency in different sexes and age groups is discussed in depth. The results are shown in Table 4. There was no statistical significance in the prevalence of zinc deficiency in different anemia states in age groups 6~<12 and 12~<18. There was also no significant difference in zinc deficiency under anemia conditions between the two age groups of the different genders assessed.

## 4. Discussion

Zinc deficiency has no characteristic signs or symptoms in metabolism or in clinic, but it can lead to widespread stunting and wasting [21]. Due to the low dietary consumption of the main sources of zinc, Asia and Africa have the highest prevalence of zinc deficiency in the global population at roughly 1 in 5 (17%) [22,23]. Estimates of child deaths due to zinc deficiency based on data from various studies range from 453,207 in the Global and Regional Child Mortality in 2004 [24] to 97,330 in the Global Burden of Disease System assessment in 2010 [25] to 116,000 in the Maternal and Child Nutrition Series in 2013 [12]. The prevalence of diseases caused by zinc deficiency is rising every year. According to studies, zinc deficiency in children is directly related to at least 50% of diarrhea-related deaths, 7% of pneumonia deaths, and 10% of malaria deaths [26]. The available data consistently show that zinc deficiency is a significant factor for population growth retardation in underdeveloped countries, even though growth retardation can occur in a variety of nutritional contexts [5]. However, clinical data in high-income environments show that infants with inadequate dietary zinc intake suffer delayed incremental growth [27]. In this study, we aimed to analyze the zinc nutritional status of children aged 6–18 in China using the latest national representative data.

Because zinc is found in the body’s numerous proteins and nucleic acids, it is difficult to measure an individual’s zinc nutritional status through laboratory tests [28]. The commonly used indicators for evaluating zinc nutritional status include serum zinc, urinary zinc, nail zinc and hair zinc [29]. It is found that serum zinc can assess the degree of risk of zinc deficiency in a specific population or subgroup by comparison [30]. A functional response to an intervention, such as growth in children or an immunological response, may be predicted using plasma zinc. With severe zinc depletion, there is a decrease in total body zinc that is reflected by the reduction in plasma zinc. In a meta-analysis of high-quality studies on the relationship between zinc consumption and plasma zinc, strong correlations were found in all groups [5]. Therefore, serum zinc is still used to evaluate the Zn nutritional status of Chinese children. 

Due to the significant effect of zinc on the growth and development of young children, current studies on zinc deficiency mainly focus on children under 5 years old. There are few studies on children over 6 years old. The prevalence of zinc deficiency varies slightly by age. In this study, the median Zn concentration of 6–18 y children was 88.39 μg/dL, and the median serum zinc in the group containing children aged 6–12 years was 88 μg/dL, which was higher than the mean Zn concentrations, 62.7 μg/dL and 72.7 μg/dL of Thai 6–13-year-old children [31] Turkish 5–13-year-old children, respectively [32]. The prevalence of Zn deficiency in this study was 9.62%. According to IZiNCG recommendations, the entire population (or population sub-group) should be regarded as being at risk of zinc insufficiency if more than 20% of the population (or population sub-group) has a serum zinc concentration below the specified cutoff [18]. Based on this standard, the overall risk of zinc deficiency in children aged 6–18 in China is quiet low. However, when compared with the 7.9% Zn deficiency in Iranian 3–18 y children [33], the prevalence in China is still higher. In studies of zinc deficiency among females aged 12 to 49 in Mexico [34] and Ecuador [35], the prevalences were even much higher, at 28% and 56%, respectively, which were already meeting the criteria for deficiency in the population. Similarly, in a study of Turkish children aged 5–16, the prevalence of zinc deficiency was found to be 27.8%. Additionally, also in a national population based study in Mexican under-12-year-old children, the prevalence of Zn deficiency reached 25.3% [36]. Compared with the above studies, the zinc deficiency in Chinese children aged 6–18 was still at a low level.

In the analysis of Zn deficiency in subgroups, we found that the prevalence of Zn deficiency in males and females was significantly different. Additionally, males had a higher risk (OR = 1.552) of inadequate Zn levels when compared to females. Prior research that evaluated zinc deficiency rarely analyzed gender differences and most focused on female participants [37,38]. In this study, although the serum zinc level of girls is lower than that of boys, the zinc deficiency rate of boys is higher than that of girls. Some studies [39,40] report that a possible reason is the difference between eating habits. Since males consume more protein than females, the serum Zn concentration is much higher than for females. Zinc intake is closely related to protein intake [41]. However, the study of Pinna et al. [42] showed that the size of the exchangeable zinc pool in the human body positively correlated with the body weight removed from fat. Compared with men, women had lower body weight and body weight removed from fat, so the serum zinc concentration was also at a lower level. In this study, men had higher serum zinc concentrations than women, but also had a higher prevalence of zinc deficiency. This is mainly because the current criteria for zinc deficiency are 74 μg/dL for men and 70 μg/dL for women.

Additionally, because of this dietary factor, there a statistical difference in the prevalence of zinc deficiency under anemia. People with anemia had a higher prevalence of Zn deficiency, 10.88%, than those who do not have anemia, 9.54%. Meanwhile, in this study, the risk of zinc deficiency in anemic people is 1.26 times higher than that of non-anemic people. Iron and zinc are both essential micronutrients for human growth and health. Deficiencies in these nutrients are very common in the population. Cross-sectional studies showed that serum zinc levels were positively correlated with markers of hemoglobin and iron status [43]. Numerous human investigations have confirmed the link between low zinc and low hemoglobin [44,45], and animal research has shown that zinc is necessary for iron metabolism [46]. In a mechanism study, zinc has been shown to induce iron uptake and transcellular transport by inducing the expression of bivalent metal iron transporter-1 (DMT1) and ferritin (FPN1) [47,48], respectively. Motadi et al. [49] found that the proportion of iron deficiency accompanied by zinc deficiency in preschool children was 2%. Studies have reported that the zinc nutritional status of preschool children is an independent positive correlation factor with their hemoglobin level, and zinc deficiency can increase the risk of anemia in this population [50]. In two other studies, plasma zinc has been shown to be a strong predictor of hemoglobin and is independent of iron status [51]. Zinc may affect hemoglobin through several zinc-dependent enzyme systems involved in hemoglobin synthesis and erythrocyte production [51] Therefore, it is possible to utilize zinc as a sign of iron deficiency anemia [52]. According to our findings and the studies reported above, we conducted a stratified discussion on the two factors of anemia and zinc deficiency in this population. Unfortunately, no meaningful results were found. Our analysis may be due to the small sample size under the second stratification, which has a great impact on the statistical results. Therefore, the discussion was not continued. However, we will continue to explore the relationship between Zn deficiency and anemia.

At the same time, the prevalence of zinc deficiency for children under different nutritional conditions is also different. Children with stunting had the highest prevalence of zinc deficiency compared with other nutritional status. Additionally, stunting is one of the risk factors of zinc deficiency. According to reports, every year, 3 million children die from undernutrition-related causes, including fetal growth restriction, stunting, wasting, and vitamin A and zinc deficiencies [12]. Zinc deficiency is closely associated with stunting. Zinc deficiency can lead to growth retardation, which can also aggravate zinc deficiency. A study in Nepal found a strong correlation between zinc and children’s stunting. Zn supplementation can reduce children’s growth retardation by 1 to 7.5 percent [53]. Trials conducted in areas with high rates of stunting and/or wasting (such as Bangladesh [54,55] and India [56]) have also shown that zinc supplementation is beneficial for the entire study population. However, in this study, we also found that children with overweight and obesity have a lower risk of zinc deficiency than the normal group. A previous study analyzed the effects of zinc supplementation on insulin resistance and metabolic syndrome in obese children. Zinc supplementation is considered to be a useful and safe additional intervention [57]. However, the effectiveness of zinc supplements in treating obesity and diabetes has not been demonstrated in large-scale studies [58]. We consider that the difference in dietary intake may lead to more protein intake, which leads to higher zinc intake. However, the specific reasons need more research to be further understood.

The differences in zinc deficiency in city type, district and income may all be caused by different economic levels and environmental, economic, and lifestyle factors, including dietary models. However, in this study, we have no way to collect and analyze environmental factors. We hypothesize that the differences in economic level and dietary patterns in different regions may be responsible for the differences in zinc deficiency rates. Studies [32] have shown that the prevalence of zinc deficiency in children in developed countries is as low as 0–2% [59,60,61], while in developing countries it is as high as 13–55.7% [62,63]. It shows that economic level has a significant influence on zinc deficiency. The results of this study further confirm this conclusion. In this study, we found that children from families with an annual income of less than CNY 7500 had a higher risk of zinc deficiency compared with other groups. At the same time, we also found that children who live in rural areas have a higher prevalence of Zn deficiency and a higher risk of inadequate Zn reservation. This is consistent with the conclusion from a prior study—that is, poor dietary habits and nutritional status can seriously affect the vitamin and mineral levels of children in rural areas [32]. Although there were no significant differences in the risk of zinc deficiency among different districts, the prevalence of zinc deficiency in central and western regions is significantly higher than that in the economically developed eastern regions. Therefore, more attention should be paid to the zinc nutritional status of children from low-income families and rural areas.

Based on the national representative data, this study is the first to thoroughly assess the nutritional status of zinc in Chinese children aged 6 to 18 years old. We also, for the first time, assessed our children’s zinc deficiency and compared it with other countries. The potential causes of zinc deficiency were also investigated. The main limitations of this study are as follows: Firstly, we could not examine the level of dietary zinc consumption for this population. Therefore, no further analysis of the relationship between dietary zinc intake and serum zinc levels was possible. The second limitation involves the criteria for determining zinc deficiency in children. The widely used criteria for determining zinc deficiency in children are based on the NHANES II and IZiNCG. However, for children under 10 years old, there are only threshold values of Zn deficiency for morning (non-fasting) and afternoon (non-fasting). There is currently no internationally recognized or nationally representative standard for fasting serum zinc deficiency in children under 10 years of age. Due to this limitation, the standard of people over 10 years old was used uniformly for both children aged 6–10 and children aged 10 and above in this study. Thirdly, in this study, we did not detect the element iron. The screening criteria for anemia are indeed based on hemoglobin. The interaction between iron and zinc will be analyzed in the follow-up study. In future studies, we will first establish zinc deficiency evaluation criteria for children aged 6–10 years old based on data from the Chinese population, and verify the existing evaluation criteria for children over 10 years old in the Chinese population. At the same time, we will also test whether zinc deficiency increases the risk of anemia in the Chinese population, and explore the combined effects of zinc and iron.

## 5. Conclusions

Currently, there are not enough studies to fully assess children’s nutritional status for zinc, particularly those between the ages of 6 and 18. However, because zinc plays such a significant role in the entire process of growth and development in children, it is crucial to completely understand the zinc nutritional status and to provide zinc supplements in a timely manner. The 64,850 6–18-year-old Chinese children from CNNHS 2016–2017 had a median Zn concentration of 88.39 μg/dL. Additionally, the prevalence of Zn deficiency was 9.62%. According to the criteria of zinc deficiency in the population (less than 20%), zinc deficiency in children aged 6–18 in China is at a low level. Possible risk factors of Zn deficiency are being male, children with anemia, stunting and low family incomes, and children who live in rural areas. More attention needs to be paid to the zinc nutritional status of children in rural areas and with low income and malnourishment. A continuous follow-up for this population is also needed.

This study not only evaluated the zinc nutritional status of children in China, but also analyzed the possible influencing factors of zinc deficiency. It provides data support for better promotion of children’s health.

## Figures and Tables

**Table 1:** Serum zinc levels of Chinese children under different stratified factors.

Indexes	N (%)	Zinc Concentration		χ^2^	*p*
		*Median* (μg/dL)	*Q*_25_~*Q*_75_ (μg/dL)		
Total	64,850 (100%)	88.39	80.00~98.80		
Sex				174.16	<0.001
Male	32,384 (49.94%)	89.00	80.30~99.60		
Female	32,466 (50.06%)	88.00	79.00~97.80		
Age group				7.13	0.008
6~<12	36,065 (55.61%)	88.00	80.00~98.20		
12~<18	28,785 (44.39%)	89.00	80.00~99.00		
Anemia				77.59	<0.001
Yes	3285 (5.07%)	86.40	78.00~96.00		
No	61,565 (94.93%)	88.84	80.00~99.00		
Nutritional status				264.32	<0.001
Stunting	890 (1.37%)	86.00 ^a^	77.00~96.00		
Wasting	5681 (8.76%)	88.00 ^b^	80.00~98.00		
Normal	45,130 (69.59%)	88.00 ^b^	79.00~98.00		
Overweight	7258 (11.19%)	90.00 ^c^	81.00~100.00		
Obesity	5885 (9.07%)	91.00 ^d^	82.00~101.00		
City type				299.18	<0.001
City	31,300 (48.27%)	89.70	81.00~99.00		
Rural	33,550 (51.73%)	87.30	78.90~98.00		
District				1475.58	<0.001
Eastern	23,510 (36.25%)	90.59 ^a^	82.00~100.60		
Central	19,461 (30.01%)	89.10 ^b^	79.86~101.80		
Western	21,879 (33.70%)	86.00 ^c^	78.00~94.00		
Income (yuan)				153.19	<0.001
<7500	5115 (7.89%)	86.00 ^a^	78.00~96.00		
7500~<12,500	5522 (8.52%)	87.00 ^a^	78.00~96.00		
12,500~<20,000	5053 (7.79%)	87.68 ^b^	79.00~97.00		
>20,000	6222 (9.59%)	89.00 ^c^	81.00~99.00		

^a,b,c,d^: The comparison of differences between groups; the same letter has no difference, different letters have differences.

**Table 2:** The prevalence of zinc deficiency in Chinese 6–18-year-old children.

Indexes	N	Zinc Deficiency			χ^2^	*p*
		n	Prevalence (%)	95%*CI*		
Total	64,850	6320	9.62	9.22~10.00		
Sex					68.26	<0.001
Male	32,384	3795	11.14	10.56~11.72		
Female	32,466	2525	7.86	7.35~8.37		
Age group					15.77	<0.001
6~<12	36,065	3638	10.44	9.85~11.02		
12~<18	28,785	2682	8.85	8.33~9.38		
Anemia					17.28	<0.001
Yes	3285	389	10.88	9.17~12.59		
No	61,565	5931	9.54	9.14~9.94		
Nutritional status					3.98	0.003
Stunting	890	123	13.55 ^a^	10.39~16.71		
Wasting	5681	568	9.11 ^b^	7.79~10.44		
Normal	45,130	4514	9.88 ^b^	9.40~10.35		
Overweight	7258	647	8.94 ^c^	7.85~10.03		
Obesity	5885	467	8.02 ^c^	6.84~9.20		
City type					47.27	<0.001
City	31,300	2634	8.20	7.69~8.71		
Rural	33,550	3686	10.92	10.34~11.51		
District					112.87	<0.001
Eastern	23,510	1683	6.15 ^a^	5.60~6.70		
Central	19,461	2250	12.36 ^b^	11.62~13.11		
Western	21,879	2387	12.12 ^b^	11.34~12.89		
Income (yuan)					15.33	<0.001
<7500	5115	580	13.11 ^a^	11.44~14.78		
7500~<12,500	5522	631	10.69 ^a^	9.21~12.17		
12,500~<20,000	5053	505	8.64 ^b^	7.29~9.99		
>20,000	6222	543	6.88 ^c^	5.88~7.89		

^a,b,c^: Horizontal comparison of differences between groups, The same letter has no difference; different letters have differences.

**Table 3:** Multivariate logistic regression model for risk factors associated with Zn deficiency.

Indexes		OR	95%CI	*p*
Sex	Female	-	-	-
	Male	1.552	1.410~1.709	<0.001
Age group	12~<18	-	-	-
	6~<12	1.068	0.974~1.171	0.159
Anemia	No	-	-	-
	Yes	1.260	1.130~1.405	<0.001
Nutritional status	Normal	-	-	-
	Stunting	1.443	1.190~1.750	<0.001
	Wasting	1.000	0.912~1.096	0.992
	Overweight	0.881	0.808~0.960	0.004
	Obesity	0.776	0.702~0.857	<0.001
City type	City	-	-	-
	Rural	1.239	1.124~1.366	<0.001
District	Eastern	-	-	-
	Central	1.090	0.971~1.224	0.304
	Western	1.079	0.964~1.207	0.478
Income (yuan)	>20,000	-	-	-
	<7500	1.261	1.095~1.453	0.017
	7500~<12,500	1.224	1.069~1.401	0.079
	12,500~<20,000	1.096	0.952~1.261	0.353

**Table 4:** Association between anemia and zinc deficiency in different age groups.

Indexes		N	Zinc Deficiency			χ^2^	*p*
			n	Prevalence (%)	95%*CI*		
Total		64,850	6320	9.62	9.22~10.00		
Sex						68.26	<0.001
Male		32,384	3795	11.14	10.56~11.72		
Female		32,466	2525	7.86	7.35~8.37		
Age group	Anemia	36,065	3638			3.54	0.06
6~<12	Yes	1419	184	13.01	10.00~16.02		
	No	34,646	3454	10.33	9.73~10.92		
	Anemia	28,785	2682			0.59	0.44
12~<18	Yes	1866	205	9.60	7.56~11.65		
	No	26,919	2477	8.80	8.26~9.34		
Sex group	Anemia					2.54	0.11
Male	Yes	1156	161	11.06	10.47~11.65		
	No	31,228	3634	13.49	10.31~16.68		
	Anemia					3.00	0.08
Female	Yes	2129	228	9.41	7.44~11.39		
	No	30,337	2297	7.74	7.21~8.27		
Male	Anemia	17,969				2.73	0.10
6~<12	Yes	685	101	12.27	11.39~13.15		
	No	17,284	2148	15.98	11.19~20.78		
	Anemia	14,415				0.02	0.89
12~<18	Yes	471	60	10.18	6.58~13.78		
	No	13,944	1486	9.92	9.13~10.71		
Female	Anemia	18,096				0.82	0.37
6~<12	Yes	734	83	9.42	6.33~12.52		
	No	17,362	1306	8.04	7.27~8.81		
	Anemia	14,370				2.69	0.10
12~<18	Yes	1395	145	9.41	6.97~11.86		
	No	12,975	991	7.46	6.73~8.18		

## Data Availability

All data for this article are from CNNHS 2016–2017 and specific data are not publicly available at this time.
